# Oxidative Stress and Inflammation Biomarker Expression in Obstructive Sleep Apnea Patients

**DOI:** 10.3390/jcm10020277

**Published:** 2021-01-13

**Authors:** Antonino Maniaci, Giannicola Iannella, Salvatore Cocuzza, Claudio Vicini, Giuseppe Magliulo, Salvatore Ferlito, Giovanni Cammaroto, Giuseppe Meccariello, Andrea De Vito, Alice Nicolai, Annalisa Pace, Marco Artico, Samanta Taurone

**Affiliations:** 1 Department of Medical and Surgical Sciences and Advanced Technologies “GF Ingrassia” ENT Section, University of Catania, 95123 Catania, Italy; tonymaniaci@hotmail.it (A.M.); s.cocuzza@unict.it (S.C.); ferlito@unict.it (S.F.); 2 Department of Sensory Organs, “Sapienza” University of Rome, 00100 Rome, Italy; giannicola.iannella@uniroma.it (G.I.); giuseppe.magliulo@uniroma1.it (G.M.); alice.nicolai@uniroma1.it (A.N.); annalisapace90@gmail.com (A.P.); samanta.taurone@uniroma1.it (S.T.); 3 Department of Head-Neck Surgery, Otolaryngology, Head-Neck and Oral Surgery Unit, Morgagni Pierantoni Hospital, 47121 Forlì, Italy; claudio@claudiovicini.com (C.V.); giovanni.cammaroto@hotmail.com (G.C.); drmeccariello@gmail.com (G.M.); 4 Department ENT and Audiology, University of Ferrara, 44121 Ferrara, Italy; 5 Department of Head-Neck Surgery, Otolaryngology, Head-Neck and Oral Surgery Unit, Ospedale “Santa Maria delle Croci”, Viale Vincenzo Randi, 5, 48121 Ravenna, Italy; dr.andrea.devito@gmail.com

**Keywords:** obstructive sleep apnea, intermittent hypoxia, cardiovascular risk, tumour necrosis factors, lipid peroxidation, cell-free DNA

## Abstract

Obstructive Sleep Apnea Syndrome (OSAS) is a respiratory sleep disorder characterised by repeated episodes of partial or complete obstruction of the upper airway during the night. This obstruction usually occurs with a reduction (hypopnea) or complete cessation (apnea) of the airflow in the upper airways with the persistence of thoracic-diaphragmatic respiratory movements. During the hypopnea/apnea events, poor alveolar ventilation reduces the oxygen saturation in the arterial blood (SaO_2_) and a gradual increase in the partial arterial pressure of carbon dioxide (PaCO_2_). The direct consequence of the intermittent hypoxia is an oxidative imbalance, with reactive oxygen species production and the inflammatory cascade’s activation with pro and anti-inflammatory cytokines growth. Tumour necrosis factors, inflammatory cytokines (IL2, IL4, IL6), lipid peroxidation, and cell-free DNA have been found to increase in OSAS patients. However, even though different risk-related markers have been described and analysed in the literature, it has not yet been clarified whether specified inflammatory bio-markers better correlates with OSAS diagnosis and its clinical evolution/comorbidities. We perform a scientific literature review to discuss inflammatory and oxidative stress biomarkers currently tested in OSAS patients and their correlation with the disease’s severity and treatment.

## 1. Introduction

Obstructive Sleep Apnea Syndrome (OSAS) is a clinical condition characterised by the occurrence, during sleep, of cyclic episodes of complete (apnea) or partial (hypopnea) obstruction of the upper airways for more than 10 s, with the persistence of thoracoabdominal movements [1]. The lack of proper nocturnal alveolar ventilation during apnea episodes usually results in a reduction in the oxygen saturation of the arterial blood (SaO_2_) and, in the case of prolonged efforts, in a gradual increase in the blood pressure of carbon dioxide (PaCO_2_) [2].

At the end of apneic events, micro awakenings occur, defined as “arousal“ an electroencephalographic alteration of the micro-and macrostructure of sleep. These nocturnal awakenings are associated with autonomic changes with the sympathetic nervous system’s activation, temporary increase in blood pressure, and tachycardia [3,4,5].

OSAS is a frequent and often underestimated disease, affecting between 2% and 4% of middle-aged women and men. However, it has been estimated in some clinical studies that it can reach a much higher incidence in older people between 20% and 60% in people over 65 years with significant difference in the Apnea-Hypopnea Index (AHI) [6,7].

As described by several authors, OSAS is associated with an increased incidence of cardiovascular diseases such as high blood pressure, ischemic heart disease, arrhythmias, and cerebrovascular problems. Its treatment with increasingly innovative methods provides promising results on reducing associated comorbidities but Continuous Positive Airway Pressure (CPAP) therapy remains a fundamental approach [8,9,10,11,12,13].

Like patients suffering from dysmetabolic syndromes, the obstructive apnea syndrome patients would seem to be characterised by a chronic systemic inflammatory state [14,15,16].

The nocturnal episodes of the upper airways collapse with consequent chronic intermittent hypoxia lead to repetitive cycles of hypoxia and reoxygenation, enhanced systemic oxidative stress and lead to the development of systemic inflammatory-related biomarkers [17,18].

Although the mechanisms linking OSAS to cardiovascular disorders remain fully elucidated, the endothelial dysfunction related to the intermittent hypoxemia and the consequent generation of reactive oxygen species (ROS) and pro-inflammatory molecules—could play an important role. Therefore, different authors hypothesised that the oxidative stress is the behind conditions in OSAS patients that could promote ischemic heart attack and other cardiovascular damages.

Reactive oxygen species (ROS) in particular, cause damage to the vascular endothelium from the early stages of the disease and stimulates the expression of the adhesion molecules of leukocytes (L-selectin, integrins) and related endothelial adhesion molecules (E-selectin, P-selectin, ICAM-1, VECAM-1) [19,20]. Endothelial lesion resulting from these biomolecular alterations, it would seem to lead to the microvascular damage of patients with OSAS [19].

The pathway consisting of reactive oxygen species induced by intermittent hypoxia (ROS) and the inducible transcription factor of hypoxia-1 (HIF-1) is responsible for deleterious cardiovascular outcomes, including aortic dissection development [21].

The resulting imbalance between oxidative stress from increased oxygen free radicals and an ineffective antioxidant capacity can be quantified through various inflammatory biomarkers, molecules generated by the oxidation of nucleic acids, proteins and lipids and expression of multilevel cell damage [22,23,24,25,26]. On the other hand, increased ROS production resulting from the hypoxia/reoxygenation cycles can increase cytokines and adhesion molecules’ expression linked to endothelial dysfunction and cardiovascular disease.

Ordinarily, oxidative stress biomarkers were defined as a large comprehensive species, including both the factors liberated as free oxygen radicals and plasma markers of systemic inflammation.

Usually, oxidative stress biomarkers have been defined as a large complete species, including both factors released as oxygen free radicals and plasma markers of systemic inflammation. However, to fully understand the physiopathogenic mechanisms of the systemic inflammatory process, the different pro-inflammatory patterns and circulating inflammatory markers must be identified and specified (Table 1).

**Figure 1:**
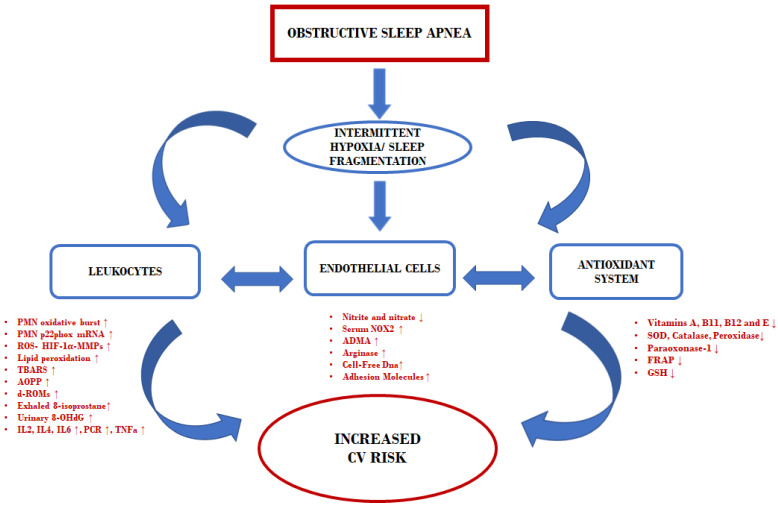
Flow chart OSAS flogosis pathway. Abbreviations: PMN oxidative burst, polymorphonuclear neutrophils; TBRSA, thiobarbituric acid reactive substances; Urinary 8-OHdG, urinary excretion of 8-hydroxy-2’-deoxyguanosine; ADMA, Asymmetric dimethylarginine; FRAP, ferric reducing antioxidant power; SOD, Superoxide dismutase; d-ROMs, reactive oxygen metabolites GSH, Glutathione; AOPP, Advanced Oxidation Protein Products; ROS, Reactive oxygen species; CV, Cardiovascular; HIF, Hypoxia-inducible factor; MMP, Matrix metalloproteinase.

Macrophages play a fundamental role in the inflammatory process and polymorphonuclear neutrophils. Through the oxidative burst process, macrophages produce a whole series of molecules and the reactive oxygen species, including substances reactive to thiobarbituric acid, 8-OHdG and asymmetric dimethylarginine (Figure 1). Therefore, it is essential to correctly discriminate between the various classes of molecules and the respective oxidative pathway involved through blood and urine assays in OSAS patients with cardiovascular disorders and metabolic diseases such as type II diabetes mellitus neurodegenerative disorders between which cognitive dysfunctions. 

This manuscript has revised the existing literature regarding oxidative stress and inflammation biomarkers expression in obstructive OSAS patients classifying the main inflammation biomarkers of a hyperexpression found in OSAS patients. This review tries to clarify what has been reported and discussed to date about the different inflammatory markers in OSAS patients.

## 2. Materials and Methods

### Study Protocol

A review of medical literature was conducted using PubMed, Cochrane, and EMBASE databases from 2000 to 2020. Two authors (A.M.) and (G.I.) independently selected articles by title, abstract and full text. The study’s inclusion was then discussed with an additional author (S.T.) to formulate a suitability judgment. Thus, we applied during the literature revision the following Inclusion criteria: full-text English articles; studies with the adult population or animals; reported values for at least one of the markers of interest. Duplicate results have been removed.

We utilized the main specific keywords in regards such as obstructive sleep apnea, oxidative stress, inflammation biomarkers, protein C reactive, tumour necrosis factor-alpha (TNF-α), interleukin 6 (IL-6), interleukin 8 (IL-8), cell-free DNA, NADPH oxidase, liperoxidation products, advanced oxidation protein products (AOPP), 8-hydroxy-2-deoxyguanosine, nitrite and nitrate, serum NOX2, Asymmetric dimethylarginine (ADMA), arginase, antioxidant system, glutathione, vitamin C and vitamin E.

## 3. Results and Discussion

The characteristic imbalance between pro-inflammatory and anti-inflammatory factors leads to increased oxidative stress, mainly due to increased oxygen free radicals and an ineffective antioxidant capacity [23,24].

The different molecules involved in chronic systemic inflammation, whose quantification is possible through various inflammatory blood or urinary biomarkers deriving from nucleic acids, proteins and lipids are described in Table 2.

The repeated cycles of chronic hypoxia/reoxygenation and sleep fragmentation that lead to increased ROS production, circulating cytokines and adhesion molecules in OSAS patients are correlated in the literature to cardiovascular, metabolic and neurodegenerative comorbidities (Table 3).

### 3.1. Oxidative Stress Markers

#### 3.1.1. Leukocytes’ Oxidative Derived

The respiratory burst or oxidative burst is responsible for producing and releasing reactive oxygen species such as superoxide anion, hydrogen peroxide, and hypochlorite anion [36]. Different triggering stimuli, including the hypoxia characteristic of the OSAS pathology, could originate the reaction [37]. This process is enzyme-mediated, specifically by nicotinamide adenine dinucleotide phosphate oxidase (NADPH oxidase), reducing free oxygen O_2_ to superoxide the subsequent cascade production of other reactive molecules such as anions hydroxide, peroxide, hypochlorite, and nitrogen monoxide [38]. Moreover, this step is precisely responsible for the oxidation of the biological compounds such as lipids, proteins and DNA and the impaired plasma concentration of associated oxidative markers [39].

Schulz et al. reported in 2000 that neutrophil superoxide generation is higher in OSAS patients than healthy subjects [27]. In particular, the comparison of superoxide release after stimulation in 18 untreated OSAS patients revealed significantly increased levels than the two control groups of 10 healthy volunteers and 10 patients without OSAS (*p* < 0.01 for each analysis).

Moreover, the authors stated that CPAP therapy reduces the superoxide release characteristic of the respiratory burst and a consequent improvement on the genesis of disorders cardiovascular [37]. Only two nights of CPAP therapy reduced superoxide release by 43% compared to the initial levels (*p* < 0.01).

#### 3.1.2. NADPH Oxidase

Numerous studies in the literature demonstrate the upregulated NADPH enzyme expression in OSAS patients’ leukocytes due to the hypoxic stimulus [41,42,43,44,45,46,47,48,49,50].

As demonstrated by Liu et al. as the expression of specific polymorphisms of the NADPH phagocytic oxidase subunit is significantly increased in OSAS patients compared to healthy subjects (*p* < 0.01) demonstrating the correlation of the gene with the pathology [51]. Likewise, polymorphism’s functional mechanisms entail pathophysiological consequences such as oxidative modifications of LDL, intimal lymphocytic infiltration, and successive formation of atherosclerosis [41,42,43].

Furthermore, experimental studies on mouse models have shown the neurobehavioral disorders and hypertension associated with OSAS and increased oxidative stress and inflammation, defining NADPH oxidase as a possible therapeutic target for the obstructive disease [44,45,46].

#### 3.1.3. Liperoxidation Products

The exponential increase in oxygen free radicals released by leukocyte cells leads to the production of biological compounds of peroxidation lipids and corresponding biomarkers.

Among the reactive substances that reflect the increase in lipid peroxidation are the thiobarbituric acid reacting substances and malonaldehyde, reactive substances are deriving from the oxidation of fatty acids with three or more double bonds [48].

As demonstrated by several literature studies, thiobarbituric acid reactive species and malondialdehyde are significantly correlated with the severity of OSAS [48,49,50,51,52,53,54].

Another marker is represented by the Isoprostanes that derive mainly arachidonic acid, considered reliable and detectable oxidative stress biomarkers. Its high concentration in cardiovascular disorders and atherosclerosis improve vasoconstrictor tone and can, therefore, contribute to higher arterial hypertension in OSAS patients [23,53,54,55,56,57,58].

In literature, it is shown how the increase of these substances causes a higher cardiovascular risk. Furthermore, CPAP treatment has proven effective in reducing the blood concentration of peroxidised lipids and the risk of associated comorbidity [48,49,59]

#### 3.1.4. Advanced Oxidation Protein Products (AOPP)

Protein oxidation products and related markers quantifiable through blood or salivary assays are used to quantify OSAS-induced oxidative stress [59,60,61,62,63,64]. These include advanced oxidation protein products (AOPP) whose correlation with oxidative stress from hypoxemia is well known in the literature [18,65,66,67,68,69].

The analysis of AOPP levels in patients with severe and moderate OSAS compared to mild and healthy is significantly higher, correlating biomarkers to the apnea-hypopnea index’s severity [62,63]. He et al. found a correlation between neurocognitive impairment in patients with moderate to severe OSAS is associated with oxidative stress expressed with AOPP biomarker [70].

Continuous positive airway pressure (CPAP) treatment has been shown to have a significant effect on serum AOPP values [60,61]. Furthermore, very strong negative correlations (*r* = −0.987, *p* < 0.001) between total antioxidant capacity and AOPP levels were demonstrated [65]

The AOPP salivary test showed similar trends to other oxidative stress biomarkers found in response to CPAP treatment with higher diagnostic values in the morning than in the evening (*p* < 0.05) [67,68].

Opposite results have been found by Mancuso et al. concerning the correlation between protein damage by AOPP levels and OSAS severity [37]. Besides, the OSAS in pregnant women plays a different role in oxidative stress than the general population, with significantly lower values in women with OSAS than in the controls group (*p*-value < 0.0001) [69].

Yağmur AR et al. investigated the correlation between the levels of advanced oxidative protein products (AOPP) and the polysomnographic parameters in OSAS patients undergoing CPAP treatment, finding significantly higher AOPP values in severe and moderate OSAS [42].

#### 3.1.5. Circulating Free DNA

In the literature, high concentrations of cell-free serum DNA have been detected in many acute inflammatory diseases such as stroke, cancer, heart attack or autoimmune diseases, and obstructive sleep apnea [70,71].

The biomarker examination expresses oxidative damage from oxygen free radicals in OSAS patients resulting from the degradation of fragmented nucleic acids and released to the blood plasma mainly as nucleosomes [72,73].

Circulating DNA free from serum cells is present in small-sized serum quantities of healthy individuals and is often quantified by sampling the b-globin gene [74].

Significantly higher outcomes were found in patients with severe and moderate OSAS than in subjects with mild or healthy OSAS [75]. Bauçà et al. demonstrated an association between AHI and dsDNA and nucleosomes’ concentration through a significant linear correlation analysis [47]. The authors showed that nucleosome and dsDNA levels were higher in OSAS patients than in the control group (1.47 ± 0.88 vs. 1.00 ± 0.33; *p* < 0.001 and 315.6 ± 78, 0 ng/mL vs. 282.6 ± 55.4 ng/mL; *p* = 0.007 respectively).

#### 3.1.6. 8-Hydroxy-2-deoxyguanosine

8-Hydroxy-2-deoxyguanosine (8-OHdG) is a product derived from the oxidation of deoxyribonucleic acid (DNA) used in the literature to evaluate oxidative stress damage [76,77,78,79,80,81,82]. Several studies have reported a correlation between the severity of OSAS and 8-OHdG, as well as the total antioxidant capacity (TAC) [68]. Moreover, urinary excretion (8-OHdG) has been positively correlated in patients with OSAS [77].

Jurado et al. provided a further demonstration of the validity of the biomarker, enrolling 46 patients with OSAS (mean ± SD AHI 49 ± 32.1) and 23 non-OSAS subjects (AHI 3 ± 0.9) [83]. The authors found a significant increase in the levels of malondialdehyde and 8-hydroxyoxiguanosine and a subsequent significant improvement in the oxidative stress of malondialdehyde (*p* = 0.001), 8-hydroxyoxiguanosine (*p* = 0.001) and carbonyl protein (*p* = 0.021).

### 3.2. Systemic Inflammation Markers and Circulating Cytokines

The interaction effect between obstructive gravity of sleep apnea syndrome (OSAS), serum levels of the different pro and anti-inflammatory cytokines (IL-6IL-10, IL-4, IL-2) or inflammation markers (protein C reactive, tumour necrosis factor-α) in literature is still debated [35,78,79]. Despite several authors reporting that patients with the combination of an acute cardiovascular event and sleep apnea have increased inflammatory markers like protein C reactive (CRP), IL1 a, IL-8, IL-6, TNF-α [35,75,80,81,82,83,84]

#### 3.2.1. Tumour Necrosis Alpha Factor (TNF-alpha)

The tumour necrosis alpha factor (TNF-alpha) is a central element in the modulation of systemic inflammation. The pro-inflammatory cytokine TNF-α promotes atherosclerosis by inducing the expression of cellular adhesion molecules that mediate adhesion of leukocytes to the vascular endothelium [83,84,85]. Circulating levels of TNF-α have been reported to correlate with signs of early atherosclerosis amongst healthy middle-aged men [86,87,88,89,90,91]. They are predictive of coronary heart disease and congestive cardiac failure. Moreover, persistently increased levels of TNF-α after myocardial infarction is predictive of future coronary events.

TNF concentration was found to be elevated in patients with OSAS compared to healthy subjects while Continuous Positive Airway Flow (CPAP) treatment is capable of normalising TNF values [84,85,86].

Vgontzas et al. reported a significant reduction in somnolence with the TNF-α etanercept receptor antagonist in a small pilot study [92]. At the same time, McNicholas et al. subsequently identified TNF-α among the various biomarkers, significantly correlated with the oxygen desaturation index in the OSAS patients [88,89].

Furthermore, mild OSAS is associated with an increase in pro-inflammatory systems and a corresponding reduction in anti-inflammatory systems, particularly IL-1 beta decreasing [89].

Arnardottir et al. demonstrated a significantly high correlation between BMI, the oxygen desaturation index, the hypoxia time and the minimum oxygen saturation (SaO_2_) of OSAS patients but not with the AHI index [87].

Oyama et al. in 2012 enrolled thirty-two patients diagnosed with OSAS in their study. They estimated oxidative markers before and after three months of CPAP therapy, finding plasma concentrations significantly decreased of tumour necrosis factor-α (*p* < 0.05), interleukin IL-6 (*p* < 0.01), IL-8 (*p* < 0.01) with CPAP therapy. However, IL-1β levels persisted unaltered (*p* = 0.42) [61].

#### 3.2.2. Protein C Reactive

The association of CRP with OSAS has been a subject of debate in recent years with differing conclusions in various studies that have explored the relationship.

Serum CRP levels, as demonstrated by a recent meta-analysis, are higher in patients with OSAS than in the healthy control group and a correlation between the highest body mass index and AHI and CRP values [80,81]. Yokoe et al. observed higher CRP levels in 30 patients with obstructive sleep apnea syndrome than in healthy subjects [36].

#### 3.2.3. Endothelial Related Markers

Endothelial dysfunction resulting from oxidative stress from hypoxemia is the crucial mechanism of many cardiovascular disorders such as atherosclerosis, hypertension and renal failure [15,47,58,64,65,91].

The damage from oxygen free radicals involves the expression of inflammatory cytokines and adhesion molecules (ICAM-1, VCAM-1, E-selectin), the infiltration of neutrophils and monocyte into the vascular wall of the inflammatory cells and the lower production of nitric oxide [18,19].

Nitric oxide or nitrogen monoxide (NO) is synthesised by a family of enzymes called NO synthase (NOS) thanks to the essential amino acid L-arginine. Of the three enzyme isoforms, the endothelial allows the vascular production of NO, mediating vasodilating effect, and platelet aggregation [9,12].

Different oxidative stress pathways can influence the production of NO, such as the synthesis of peroxynitrite by interaction with superoxide or the action of dimethylarginine (ADMA). It interferes with the formation of NO at high levels, the activity of the enzyme dimethylarginine dimethylaminohydrolase is reduced, leading to higher ADMA levels [13,92,93].

The quantification of NO is possible by indirect testing of its oxidative derivatives such as nitrite and nitrate [10,11]. The marker of endothelial apoptosis (CEC) represents a direct sign of cell death induced by various cardiovascular disorders and oxidative stress, at a high concentration in subjects suffering from OSAS compared to healthy subjects and sensitive to medical treatment with CPAP [17].

### 3.3. Antioxidant System Impairment

The antioxidant system constitutes a set of endogenous defence mechanisms of the organism capable of protecting against related radical damage, constituted by types of antioxidants such as enzymes such as superoxide dismutase, catalase, peroxidase or molecules such as glutathione, vitamin C and vitamin E [23,72].

OSAS patients present an imbalance between the production of oxidative agents and the balancing performed by the antioxidant system defined as total antioxidant capacity (TAC) [24,25].

Superoxide is a fundamental cellular oxidising agent whose dismutation is catalysed by the family of superoxide dismutase (SOD), a series of metallic enzymes. The enzyme allows the dissociation of the superoxide anion into molecular oxygen and hydrogen peroxide in health patients while in OSAS patients have been described lower plasma levels (SOD) [94,95].

Molecules such as glutathione, vitamin C, and vitamin E contribute to improving the state of oxidative stress in patients with OSAS, as shown in the literature in association with CPAP therapy [96,97]. Moreover, oxidative stress contributes to sleep behaviour in patients with OSAS, and the intake of antioxidants improves sleep quality in them [98,99].

Sales et al. found decreased antioxidants in patients with OSAS, suggesting a correlation between antioxidant and neuropsychological alterations in obstructive sleep apnea.

In particular, they observed decreased vitamin E (*p* < 0.006), superoxide dismutase (*p* < 0.001) and vitamin B11 (*p* < 0.001) whereas increased homocysteine levels (*p* < 0.02).

### 3.4. Physiopathological Features and Inflammatory Profiles

The association of obstructive sleep apnea (OSAS) and various cardiovascular disease forms, including hypertension, stroke, heart failure (HF), coronary heart disease, and atrial fibrillation is well established in the literature [100,101,102,103,104,105].

Peppard et al. in a cross-sectional study reported a total of 1,023/ 6424 participants (16%) with at least one manifestation of cardiovascular disease, in particular with heart failure and stroke (HR 2.38, CI:1.22–4.62; HR 1.58, CI 1.02–2.46) [106].

Moreover, Johnson et al. in a meta-analysis of 2,343 ischemic or hemorrhagic stroke and TIA patients, found patients with recurrent strokes had a higher rate of sleep breathing disorder (AHI > 10) than initial strokes (74% versus 57%, *p* = 0.013) [104]. Several authors have recently suggested that OSA / IH mechanisms that induce oxidative stress and inflammation through repeated cycles of upper airway collapse and consequent increase in sympathetic activity could lead to augmented chemoreflex [107,108,109,110,111,112,113]. Nanduri et al. proposed in 2017 an experimental protocol on rodents exposed to chronic intermittent hypoxia to simulate the alterations in oxygen saturation during obstructive sleep apnea [110]. The authors, analysing the molecular mechanisms related to long-term chronic intermittent hypoxia, revealed increased DNA methylation of the genes encoding for antioxidant enzymes and how treatment with decitabine normalised ROS levels as well as reflex chemosensory and blood pressure. The same authors have focused on the role of inducible factors linked to intermittent hypoxia in OSAS patients with comorbidities such as hypertension and type 2 diabetes. Not only the chronic intermittent hypoxia correlated with an increase in HIF-1α protein and a decrease in HIF-2α, but the increased ROS themselves provided to stimulate chemoreflex and the sympathetic nervous system with hypertension [111].

However, despite obesity and hypertension are comorbidities associated with augmented chemoreflex patients, the CPAP treatment has not demonstrated significant results on the sympathetic system activity [19,20,21,22,23,24,25,26,27,28,29,30,31,32,33,34,35,36,37,38,39,40,41,42,43,44,45,46,47,48,49,50,51,52,53,54,55,56,57,58,59,60,61,62,63,64,65,66,67,68,69,70,71,72,73,74,75,76,77,78,79,80,81,82,83,84,85,86,87,88,89,90,91,92,93,94,95,96,97,98,99,100,101,102,103,104,105,106,107,108,109,110,111,112,113,114].

Liu et al. in 2016 demonstrated in a meta-analysis the significant blood pressure reduction, both diurnal and a mean nocturnal diastolic blood pressure in 457 total patients treated with CPAP [115]. CPAP therapy in sleep apnea has also been shown to effectively reduce the patients’ sympathetic nervous system’s action and, therefore, the proarrhythmic activity [115,116,117,118].

Furthermore, Bradley et al. reported a reduction in mortality among patients who achieved a significant improvement in AHI < 15 events after CPAP [119].

A further characteristic of sleep breathing disorders is the sleep fragmentation which induces an alteration of phlogosis markers, including TNF-α [117,118]. Kaushal et al. reported in KO mice, a linear correlation between sleep fragmentation and higher circulating TNF-α values, despite preserved sleep duration [120].

In this regard, Wang et al. analysed the relationship between obstructive sleep apnea and the status of endothelial progenitor cells (EPCs), premature circulating cells reduced in both number and function in OSAS patients [121]. The authors also reported how CPAP therapy could affect EPCs by reducing systemic inflammation and sympathetic activation.

Although there are countless studies on oxidative stress in patients with obstructive sleep apneas in the literature, few authors analysed the role of inflammatory mediators in OSAS understood as a systemic inflammatory disease. In scientific studies among the oxidative stress biomarkers, those most analysed are protein C reactive (CRP), IL-6 o TNF-α [84,85,86,90].

Among the most debated topics in the literature is the correlation between systemic inflammation and cardiovascular comorbidity, the prevalence of which is higher in OSAS patients [19]. Li et al. analysed how through the study of 58 patients with obstructive sleep apnea, assessed objective and subjective daytime sleepiness concerning pro-inflammatory cytokines, demonstrating a high association with interleukin-6 (IL-6) [122].

Furthermore, as demonstrated by Kritikou et al., men with OSAS have increased CRP, IL-6, insulin resistance and leptin values compared to healthy patients [123].

However, discordant parameters were obtained after using CPAP for two months, showing no change in reducing these oxidative stress biomarkers.

Another relevant finding has been reported by Gottlieb et al., who compared the effects of CPAP treatment on OSAS patients over 12 weeks with decreasing plasma levels of protein C reactive [124]. However, while CPAP did not cause a decrease in CRP on its own, weight loss did change blood levels.

In 2015 an interesting multicentre study was carried out on obstructive sleep apnea and cardiovascular complications [125]. Analysis of the effect of CPAP treatment for 6 months in 391 patients, compared to non-use of therapy, showed no significant changes in interleukin 6 (IL-6), IL-10, protein C reactive, tumour necrosis factor (all *p* values > 0.05).

Furthermore, very contrasting data are present in the literature on oxidative stress biomarkers’ role, often validated through comparative studies on the consequences of treatment on these molecules’ serum levels.

Another phenomenon capable of reducing obstructive apneas in OSAS patients is that both weight loss and moderate-intensity aerobic exercise have been ascertained.

Just Borges et al. studied 39 patients diagnosed with OSAS by subjecting them to exercise and CPAP treatment [18]. Nevertheless, these measures have not effectively reduced oxidative stress measured through inflammatory profiles to oxidate lipids and proteins, pro and anti-inflammatory cytokines or circulating free DNA levels. Paradoxically, the levels of AOPP and IL-17A in individuals undergoing CPAP without the humidifier have increased.

Rodriguez et al. have recently conducted a multicentre and randomised study analysing different biomarkers of inflammation in 247 women diagnosed with moderate-severe OSAS [89]. They evaluated the effects of ventilatory treatment with CPAP compared to conservative therapy on tumour necrosis factor α (TNFα), interleukin 6 (IL-6), protein C reactive (CRP), brain-derived neurotrophic factor, intercellular adhesion molecule 1 (ICAM-1), superoxide dismutase (SOD) and catalase (CAT). They observed that after 12 weeks of follow-up, there had been no changes between the study group and the control group in any of the oxidative stress biomarkers evaluated. However, in women with CPAP use at least 5 h per night, TNFα levels decreased compared to the control group.

The evaluation of the new biomarker consisting of circulating endothelial cell levels (CEC) is an innovative technique that allows direct information on endothelial damage in the OSAS patient. The increase in circulating endothelial cells occurs typically in other pathologies, such as typing in myocardial injury and atherosclerotic peripheral vascular disease [126].

Endothelial impairment is a linking mechanism between obstructive sleep apnea and cardiovascular disease. The profiles of endothelial microparticles and endothelial progenitor cells reflect the degree of impairment of the endothelium, correlating with the severity of OSAS [127,128,129]. In this regard, Yun et al. recruited 104 patients by dividing them into two groups based on the diagnosis of OSAS and measured the change in the index of endothelial progenitor cells and microparticles after 4–6 weeks of CPAP therapy [127].

Another essential index was the intimate-average carotid thickness (IMT) as a marker of atherosclerosis. The analysis of the data obtained showed higher endothelial damage indices in OSAS subjects compared to non-OSAS questions. In contrast, the carotid IMT index was correlated with the severity of OSAS. Therefore, this study showed how OSAS leads to an increase in endothelial microparticles related to obstructive apnea but only partially responsive to treatment.

## 4. Data Limitations

Although several studies in the literature report a significant correlation between OSAS and numerous inflammatory biomarkers, other authors affirm conflicting results especially in the results after antioxidant therapy or OSA treatment (several authors reported similar inflammatory biomarkers’ value after positive airway pressure (PAP) therapy). A further contrasting aspect is represented by choosing the most suitable inflammatory marker among those available today, both as a direct inflammatory index and to test the body’s antioxidant capacity. These problems are also exacerbated by the typology of studies in the literature on this subject. Some authors have chosen retrospective study designs among the analyses reported, certainly less reliable than prospective ones. They also did not correlate the group of patients analysed with a control group, or the experimental antioxidant therapeutic results were tested on the animal but not human subjects.

## 5. Conclusions

Obstructive sleep apnea syndrome is a widely diffused disease strictly interconnected through the chronic systemic inflammatory substrate to different cardiovascular, metabolic and neurodegenerative comorbidities. The two fundamental pathophysiological mechanisms represented by chronic intermittent hypoxia and sleep fragmentation interact variably with the immune system triggering pro-inflammatory pathways, lymphocyte cells, monocytes up to endothelial cells.

Monitoring the circulating levels of countless inflammation markers plays a crucial role in the early identification of associated systemic risk, including the development of cardiovascular diseases. Furthermore, as expressed in the literature, the same treatment with continuous positive airway pressure in OSAS patients could improve inflammatory markers. However, some confounding factors can sometimes affect the outcomes obtained. Through a critical analysis of the underlying mechanisms and possible therapeutic implications, new approaches to the patient with sleep apnea syndrome will emerge in the future.

## Figures and Tables

**Table 1:** Main classifications of obstructive sleep apnea syndrome OSAS-related immunophlogystic biomarkers.

OXIDATIVE STRESS MARKERS	Reactive Oxygen Species (Peroxides, Superoxide, Hydroxyl Radical)
	Nicotinamide adenine dinucleotide phosphate oxidase (NADPH)
	Liperoxidation Products
	Advanced Oxidation Protein Products (AOPPs)
	Circulating free DNA
	8-Hydroxy-2-deoxyguanosine
SYSTEMIC INFLAMMATION MARKERS AND CIRCULATING CYTOKINES	IL2, IL4, IL6
	Tumour necrosis alpha factor (TNF-alpha)
	Protein C reactive
	Endothelial related markers (E-selectin, P-selectin, ICAM-1, VECAM-1)
ANTIOXIDANT SYSTEM	Superoxide dismutase, peroxidase, catalase
	Glutathione, Ferric reducing/antioxidant power (FRAP)
	Vitamin C
	Vitamin E Vitamin B11, B12

**Table 2:** Main biomarkers analysed in the literature divided by physiopathogenetic clusters.

Leukocytes’ Oxidative Derived		
Authors	Study Features	Outcomes Observed
R Schulz, S Mahmoudi, K Hattarm et al. (2000) [27]	18 OSAS patients compared vs. two control groups of 10 healthy volunteers and 10 patients without OSAS	↑ Superoxide Release markedly increased for each comparison (*p* < 0.01)
Liu HG, Zhou YN, Liu K et al. (2010) [28]	30 OSAS patients vs. 23 healthy controls	↑ NADPH oxidase p22phox mRNA in sputum samples was significantly higher in OSAS (*p* < 0.05).
E Hopps, B Canino, V Calandrino et al. (2014) [29]	48 patients with OSAS, subdivided into two subgroups: Low 21 subjects (AHI < 30) High 27 subjects (AHI > 30)	↑ TBARS and AHI value (r = 0.88, *p* < 0.0001) ↑ TBARS and ODI (r = 0.88, *p* < 0.0001)
Lavie L, Vishnevsky A, Lavie P (2004) [30]	114 patients with OSAS (55 without CVD and 59 with CVD) vs. 30 non-apneic controls.	↑ TBARS and Peroxides higher in the morning than in controls and positively correlated with RDI (*p* < 0.01)
Alzoghaibi MA, Bahammam SA (2005) [31]	34 hypertensive patients with severe obstructive sleep apnea syndrome (OSAS).	= SOD concentrations unchanged after CPAP treatment (0.22 ± 0.09 vs. 0.22 ± 0. U/mL). ↓ TBARS levels after CPAP treatment (2.81 ± 0.27 vs. 2.47 ± 0.35 mmol/mL, respectively, *p* < 0.005).
Ntalapascha M, Makris D, Kyparos A et al. (2012) [24]	18 patients with severe OSAS and 13 controls included in the study.	↑ GSH/GSSG overnight ratio and GSH significantly different than controls (*p* = 0.03 and *p* = 0.048). = Plasma protein carbonyls, erythrocyte catalase activity, 8-isoprostane, SOD, TBARS, and TAC plasma values not different (*p* > 0.05).
**Systemic Inflammation Markers and Circulating Cytokines**		
Ifergane G, Ovanyan A, Toledano R et al. (2016) [32]	43 patients with acute stroke and sleep apnea	↑ correlation between AHI, IL-6 (ρ = 0.37, *p* = 0.02) and PAI-1 (ρ = 0.31, *p* = 0.07).
Lin CC, Liaw SF, Chiu CH et al. (2016) [33]	35 patients with moderately severe to severe OSAS vs. 20 healthy controls	↓ SIRT1 was lower (*p* < 0.01) ↑ TNF-α was higher (*p* < 0.01)
Volná J, Kemlink D, Kalousová M et al. (2011) [34]	51 patients suspected for OSAS included	↑ hsCRP and ODI (R = 0.450, *p* = 0.001) ↑ hsCRP AHI (R = 0.479, *p* = 0.001) ↑ hsCRP SpO_2_ < 90 (R = 0.480, *p* = 0.001).
Wu MF, Chen YH, Chen HC et al. (2020) [35]	100 patients included in the final analysis (63 Normal to Moderate OSAS while 37Severe OSAS)	↑IL-6 level for all OSAS severity and sex had an interaction effect on (*p* = 0.030). ↑ CRP (*p* = 0.001) and ↑ IL-6 (*p* = 0.000) levels were higher in the obese group than in the non-obese group independently of OSAS severity and sex.
Yokoe T, Minoguchi K, Matsuo H, et al. (2003) [36]	30 patients with OSAS and 14 obese control subjects.	↑ Levels of CRP significantly higher in patients with OSAS than in the control group (*p* < 0.001) ↑ IL-6 significantly higher in patients with OSAS than in the control group (*p* < 0.05)
**Antioxidant System Impairment**		
Mancuso M, Bonanni E, Lo Gerfo A et al. (2012) [37]	41 untreated patients with a new diagnosis of OSAS vs. 32 healthy subjects	↑AOPP higher than in controls (293.4 ± 109.7 mmol/L vs. 203.2 ± 45.2 mmol/L; (*p* < 0.0005) ↓ FRAP lower (95% CI for the mean 0.518–0.579 mmol/L vs. 0.713–0.875 mmol/L; *p* < 0.0001). ↓ Total GSH lower (95% CI for the mean 0.389–0.449 nmol/μL vs. 0.574–0.713 nmol/μL; (*p* < 0.0001).
Katsoulis K, Kontakiotis T, Spanogiannis D et al. (2011) [38]	32 OSAS patients without comorbidities	↓ TAS significantly decreased compared with the measurement before (1.68 ± 0.11 vs. 1.61 ± 0.10 mmol/l, *p* < 0.01).
Simiakakis M, Kapsimalis F, Chaligiannis E et al. (2012) [39]	66 total subjects referred (42 patients with OSAS vs. 24 controls)	↑The levels of d-ROMS were significantly higher (*p* = 0.005) in the control group ↓ levels of antioxidant capacity in OSAS patients significantly lower (*p* = 0.004).
Sales LV, Bruin VM, D’Almeida V, et al. (2013) [40]	14 patients with obstructive sleep apnea vs. 13 controls	↓ vitamin E lower levels of (*p* < 0.006) ↓ superoxide dismutase (*p* < 0.001) ↓ vitamin B11 (*p* < 0.001) ↑ homocysteine higher concentrations (*p* < 0.02) = Serum concentrations of vitamin C, catalase, glutathione and vitamin B12 unaltered.

Abbreviations: TBARS, thiobarbituric reactive substances; SOD, Superoxide dismutase; GSH, Glutathione reduced; GSSG, Glutathione oxidised; SIRT1, Sirtuin 1; hsCRP, high sensitive protein C reactive; FRAP, ferric reducing antioxidant power; d-ROMs, reactive oxygen metabolites; OSAS, Obstructive sleep apnea syndrome; CVD, Cardiovascular disease; RDI, Respiratory Disorder Index; CPAP, Continuous Positive Airway Pressure; TAC, Total antioxidative capacity; AHI, Apnea-Hypopnea Index; TNF, Tumor Necrosis Factor; CRP, Protein C reactive; AOPP, Advanced Oxidation Protein Products; TAS, Total antioxidant status.

**Table 3:** Response to CPAP treatment on Oxidative Stress Biomarkers in Prospective Studies. Abbreviations: NS, not specified.

References	Study Design	Features	Pretreatment Scores	Post Treatment Outcomes
Del Ben et al. 2012 [41]	Prospective Study	Patients *n* = 138 (47diagnosed with primary snoring and 91 OSAS) Serum levels of soluble NOX2-derived peptide urinary 8-iso-PGF2α	Severe OSAS patients: sNOX2-dp (pg/mL) ↑ but NS Serum NOx (uM/mL) ↑ but NS urinary 8-iso-PGF2α ↑ *p* < 0.001	Severe OSAS patients after CPAP: Urinary 8-iso-PGF2α (pg/mL) ↓ *p* = 0.007 sNOX2-dp (pg/mL) ↓ *p* = 0.003 Serum NOx (uM/mL) ↓ but NS
Yagmur et al. 2020 [42]	Prospective Study	Patients *n* = 165 (125 diagnosed with OSAS, 40 control group)	AOPP: Severe OSAS vs. Mild ↑ *p* < 0.05 Severe OSAS vs. Control ↑ *p* < 0.05	AOPP ↓ *p* = 0.36 but NS
Mancuso et al. 2012 [37]	Prospective Study	Patients *n* = 73 (41 diagnosed with OSAS, 32 control group)	- AOPP ↑ *p* <0.0005 - Ferric reducing antioxidant power (FRAP) ↓ *p* < 0.0001 Total glutathione (GSH) *p* < 0.0001	AOPP unchanged NS FRAP levels ↑ *p* < 0.005 GSH not been re-evaluated
Celec et al. 2012 [43]	Prospective Study	Patients *n* = 89 diagnosed with OSAS TBARS AOPP Carbonyl stress (AGEs) Total antioxidant capacity (TAC)	TBARS AOPP Carbonyl stress (AGEs) Total antioxidant capacity (TAC)	TBARS ↓ *p* < 0.03 AOPP ↓ but NS Carbonyl stress (AGEs) ↓*p* < 0.02). Total antioxidant capacity (TAC) NS
Ye L et al. 2010 [44]	Prospective Study	Patients *n* = 179 (127 diagnosed with OSAS, 52 control group)	Serum DNA (ng/mL) ↑ *p* < 0.01 malonaldehyde (MDA) (nmol/mL) ↑ *p* < 0.01 IL-6 (pg/mL) ↑ *p* < 0.01	Serum DNA (ng/mL) ↓ *p* < 0.01 malonaldehyde (MDA) (nmol/mL) ↓ *p*= 0.04 IL-6 (pg/mL) ↓*p* < 0.01
Muñoz-Hernandez et al. 2015 [45]	Prospective Study	Patients *n* = 30 diagnosed with OSAS, no control group.	cell free-DNA = 187.93 ± 115.81 ng/mL	cell free-DNA (121.28 ± 78.98 ng/mL) ↓ *p* < 0.01
Karamanlı et al. 2014 [46]	Prospective Study	Patients *n* = 35 diagnosed with OSAS and treated with CPAP, no control group.	Nitrotyrosine 17.3 ± 30.7 pg/mL IL-6 1.1 ± 2.3 pg/mL TNF-α 28.9 ± 1.35 pg/mL 8-Isoprostane 5.7 ± 7.9 pg/mL CRP 8.3 ± 8.5 mg/l	Nitrotyrosine 4.6 ± 3.4 ↓ *p* = 0.037 IL-6 0.3 ± 0.2 ↓ *p* = 0.000 TNF-α 26.8 ± 1.9 ↓ *p* = 0.000 8-Isoprostane 3.0 ± 1.6 ↓ *p* = 0.027 CRP 6.2 ± 4.3 ↓ *p* = 0.064

## Data Availability

The data presented in this study are openly available.

## References

[1] Kapur V.K., Auckley D.H., Chowdhuri S., Kuhlmann D.C., Mehra R., Ramar K., Harrod C.G. (2017). Clinical Practice Guideline for Diagnostic Testing for Adult Obstructive Sleep Apnea: An American Academy of Sleep Medicine Clinical Practice Guideline. J. Clin. Sleep Med..

[2] Paruthi S., Rosen C.L., Wang R., Weng J., Marcus C.L., Chervin R.D., Stanley J.J., Katz E.S., Amin R., Redline S. (2015). End-Tidal Carbon Dioxide Measurement during Pediatric Polysomnography: Signal Quality, Association with Apnea Severity, and Prediction of Neurobehavioral Outcomes. Sleep.

[3] Cuspidi C., Tadic M., Sala C., Gherbesi E., Grassi G., Mancia G. (2019). Blood Pressure Non-Dipping and Obstructive Sleep Apnea Syndrome: A Meta-Analysis. J. Clin. Med..

[4] Drager L.F., McEvoy R.D., Barbe F., Lorenzi-Filho G., Redline S. (2017). INCOSACT Initiative (International Collaboration of Sleep Apnea Cardiovascular Trialists). Sleep Apnea and Cardiovascular Disease: Lessons from Recent Trials and Need for Team Science. Circulation.

[5] Iannella G., Maniaci A., Magliulo G., Cocuzza S., La Mantia I., Cammaroto G., Greco A., Vicini C. (2020). Current challenges in the diagnosis and treatment of obstructive sleep apnea syndrome in the elderly [published online ahead of print, 2020 Apr 6]. Pol. Arch. Intern. Med..

[6] Vicini C., De Vito A., Iannella G., Gobbi R., Corso R.M., Montevecchi F., Polimeni A., De Vincentiis M., Meccariello G., D’Agostino G. (2018). The aging effect on upper airways collapse of patients with obstructive sleep apnea syndrome. Eur. Arch. Otorhinolaryngol..

[7] Iannella G., Vicini C., Colizza A., Meccariello G., Polimeni A., Greco A., de Vincentiis M., de Vito A., Cammaroto G., Gobbi R. (2019). Aging effect on sleepiness and apneas severity in patients with obstructive sleep apnea syndrome: A meta-analysis study. Eur. Arch. Otorhinolaryngol..

[8] Baratta F., Pastori D., Fabiani M., Fabiani V., Ceci F., Lillo R., Lolli V., Brunori M., Pannitteri G., Cravotto E. (2018). Severity of OSAS, CPAP and cardiovascular events: A follow-up study. Eur. J. Clin. Investig..

[9] Bradley T.D., Floras J.S. (2009). Obstructive sleep apnoea and its cardiovascular consequences. Lancet.

[10] Vanek J., Prasko J., Genzor S., Ociskova M., Kantor K., Holubova M., Slepecky M., Nesnidal V., Kolek A., Sova M. (2020). Obstructive sleep apnea, depression and cognitive impairment [published online ahead of print, 2020 Mar 23]. Sleep Med..

[11] Di Luca M., Iannella G., Montevecchi F., Magliulo G., De Vito A., Cocuzza S., Maniaci A., Meccariello G., Cammaroto G., Sgarzani R. (2020). use of the transoral robotic surgery to treat patients with recurrent lingual tonsillitis. Int. J. Med. Robot. Comput. Assist. Surg..

[12] Iannella G., Magliulo G., Di Luca M., De Vito A., Meccariello G., Cammaroto G., Pelucchi S., Bonsembiante A., Maniaci A., Vicini C. (2020). Lateral pharyngoplasty techniques for obstructive sleep apnea syndrome: A comparative experimental stress test of two different techniques. Eur. Arch. Otorhinolaryngol..

[13] Iannella G., Vallicelli B., Magliulo G., Cammaroto G., Meccariello G., De Vito A., Greco A., Pelucchi S., Sgarzani R., Corso R. (2020). Long-Term Subjective Outcomes of Barbed Reposition Pharyngoplasty for Obstructive Sleep Apnea Syndrome Treatment. Int. J. Environ. Res. Public Heal..

[14] Vgontzas A.N., Bixler E.O., Chrousos G.P. (2005). Sleep apnea is a manifestation of the metabolic syndrome. Sleep Med. Rev..

[15] Alves E., Ackel-D’Elia C., Luz G.P., Cunha T.C., Carneiro G., Tufik S., Bittencourt L.R., de Mello M.T. (2012). Does physical exercise reduce excessive daytime sleepiness by improving inflammatory profiles in obstructive sleep apnea patients?. Sleep Breath..

[16] Pace A., Iannella G., Rossetti V., Visconti I.C., Gulotta G., Cavaliere C., De Vito A., Maniaci A., Cocuzza S., Magliulo G. (2020). Diagnosis of Obstructive Sleep Apnea in Patients with Allergic and Non-Allergic Rhinitis. Medicina.

[17] Kheirandish-Gozal L., Gozal D. (2019). Obstructive Sleep Apnea and Inflammation: Proof of Concept Based on Two Illustrative Cytokines. Int. J. Mol. Sci..

[18] Borges Y.G., Cipriano L., Aires R., Zovico P., Campos F.V., de Araújo M., Gouvea S.A. (2019). Oxidative stress and inflammatory profiles in obstructive sleep apnea: Are short-term CPAP or aerobic exercise therapies effective?. Sleep Breath..

[19] Wang F., Liu Y., Xu H., Qian Y., Zou J., Yi H., Guan J., Yin S. (2019). Association between Upper-airway Surgery and Ameliorative Risk Markers of Endothelial Function in Obstructive Sleep Apnea. Sci. Rep..

[20] Lino D., Freitas I.A., Meneses G.C., Martins A., Daher E.F., Rocha J., Silva Junior G.B. (2019). Interleukin-6 and adhesion molecules VCAM-1 and ICAM-1 as biomarkers of post-acute myocardial infarction heart failure. Braz. J. Med. Biol. Res..

[21] Liu W., Zhang W., Wang T., Wu J., Zhong X., Gao K., Liu Y., He X., Zhou Y., Wang H. (2019). Obstructive sleep apnea syndrome promotes the progression of aortic dissection via a ROS- HIF-1α-MMPs associated pathway. Int. J. Biol. Sci..

[22] Düger M., Seyhan E.C., Günlüoğlu M.Z., Bolatkale M., Ozgul M.A., Turan D., Uğur E., Ülfer G. (2020). Does ischemia-modified albumin level predict severity of obstructive sleep apnea?. Sleep Breath..

[23] Passali D., Corallo G., Yaremchuk S., Longini M., Proietti F., Passali G.C., Bellussi L. (2015). Oxidative stress in patients with obstructive sleep apnoea syndrome. Acta Otorhinolaryngol. Ital..

[24] Ntalapascha M., Makris D., Kyparos A., Tsilioni I., Kostikas K., Gourgoulianis K., Kouretas D., Zakynthinos E. (2012). Oxidative stress in patients with obstructive sleep apnea syndrome. Sleep Breath..

[25] Lira A.B., de Sousa Rodrigues C.F. (2016). Evaluation of oxidative stress markers in obstructive sleep apnea syndrome and additional antioxidant therapy: A review article. Sleep Breath..

[26] Franco C.M.R., Lima A.M.J., Ataide L., Lins O.G., Castro C.M.M., Bezerra A.A., de Oliveira M.F., Oliveira J.R.M. (2012). Obstructive sleep apnea severity correlates with cellular and plasma oxidative stress parameters and affective symptoms. J. Mol. Neurosci..

[27] Schulz R., Mahmoudi S., Hattar K., Sibelius U., Olschewski H., Mayer K., Seeger W., Grimminger F. (2000). Enhanced release of superoxide from polymorphonuclear neutrophils in obstructive sleep apnea. Impact of continuous positive airway pressure therapy. Am. J. Respir. Crit. Care Med..

[28] Liu H.G., Zhou Y.N., Liu K., Xu Y.J. (2010). Nicotinamide-adenine dinucleotide phosphate oxidase p22phox expression in induced sputum cells for patients with obstructive sleep apnea hypopnea syndrome. Chin. J. Tuberc. Respir. Dis..

[29] Hopps E., Canino B., Calandrino V., Montana M., Lo Presti R., Caimi G. (2014). Lipid peroxidation and protein oxidation are related to the severity of OSAS. Eur. Rev. Med. Pharmacol. Sci..

[30] Biller J.D., Takahashi L.S. (2018). Oxidative stress and fish immune system: Phagocytosis and leukocyte respiratory burst activity. An. Acad. Bras. Ciências.

[31] Alzoghaibi M.A., Bahammam A.S. (2005). Lipid peroxides, superoxide dismutase and circulating IL-8 and GCP-2 in patients with severe obstructive sleep apnea: A pilot study. Sleep Breath..

[32] Ifergane G., Ovanyan A., Toledano R., Goldbart A., Abu-Salame I., Tal A., Stavsky M., Novack V. (2016). Obstructive Sleep Apnea in Acute Stroke: A Role for Systemic Inflammation. Stroke.

[33] Lin C.C., Liaw S.F., Chiu C.H., Chen W.J., Lin M.W., Chang F.T. (2016). Effects of nasal CPAP on exhaled SIRT1 and tumor necrosis factor-α in patients with obstructive sleep apnea. Respir. Physiol. Neurobiol..

[34] Volná J., Kemlink D., Kalousová M., Vávrová J., Majerová V., Mestek O., Svarcová J., Sonka K., Zima T. (2011). Biochemical oxidative stress-related markers in patients with obstructive sleep apnea. Med. Sci. Monit..

[35] Wu M.-F., Chen Y.-H., Chen H.-C., Huang W.-C. (2020). Interactions among Obstructive Sleep Apnea Syndrome Severity, Sex, and Obesity on Circulatory Inflammatory Biomarkers in Patients with Suspected Obstructive Sleep Apnea Syndrome: A Retrospective, Cross-Sectional Study. Int. J. Environ. Res. Public Heal..

[36] Yokoe T., Minoguchi K., Matsuo H., Oda N., Minoguchi H., Yoshino G., Hirano T., Adachi M. (2003). Elevated levels of C-reactive protein and interleukin-6 in patients with obstructive sleep apnea syndrome are decreased by nasal continuous positive airway pressure. Circulation.

[37] Mancuso M., Bonanni E., LoGerfo A., Orsucci D., Maestri M., Chico L., DiCoscio E., Fabbrini M., Siciliano G., Murri L. (2012). Oxidative stress biomarkers in patients with untreated obstructive sleep apnea syndrome. Sleep Med..

[38] Katsoulis K., Kontakiotis T., Spanogiannis D., Vlachogiannis E., Kougioulis M., Gerou S., Daskalopoulou E. (2011). Total antioxidant status in patients with obstructive sleep apnea without comorbidities: The role of the severity of the disease. Sleep Breath..

[39] Simiakakis M., Kapsimalis F., Chaligiannis E., Loukides S., Sitaras N., Alchanatis M. (2012). Lack of effect of sleep apnea on oxidative stress in obstructive sleep apnea syndrome (OSAS) patients. PLOS ONE.

[40] Sales L.V., Bruin V.M., D’Almeida V., Pompéia S., Bueno O.F., Tufik S., Bittencourt L. (2013). Cognition and biomarkers of oxidative stress in obstructive sleep apnea. Clinics.

[41] Del Ben M., Fabiani M., Loffredo L., Polimeni L., Carnevale R., Baratta F., Brunori M., Albanese F., Augelletti T., Violi F. (2012). Oxidative stress mediated arterial dysfunction in patients with obstructive sleep apnoea and the effect of continuous positive airway pressure treatment. BMC Pulm. Med..

[42] Yağmur A.R., Çetin M.A., Karakurt S.E., Turhan T., Dere H.H. (2020). The levels of advanced oxidation protein products in patients with obstructive sleep apnea syndrome. Ir. J. Med. Sci..

[43] Celec P., Hodosy J., Behuliak M., Pálffy R., Gardlik R., Halčák L., Mucska I. (2011). Oxidative and carbonyl stress in patients with obstructive sleep apnea treated with continuous positive airway pressure. Sleep Breath..

[44] Ye L., Ma G.H., Chen L., Li M., Liu J.L., Yang K., Li Q.Y., Li N., Wan H.Y. (2010). Quantification of circulating cell-free DNA in the serum of patients with obstructive sleep apnea-hypopnea syndrome. Lung.

[45] Muñoz-Hernandez R., Vallejo-Vaz A.J., ArmengolÁngeles S., Moreno-Luna R., Caballero-Eraso C., Macher H.C., Villar J., Merino A.M., Castell J., Capote F. (2015). Obstructive Sleep Apnoea Syndrome, Endothelial Function and Markers of Endothelialization. Changes after CPAP. PLoS ONE.

[46] Karamanlı H., Özol D., Ugur K.S., Yıldırım Z., Armutçu F., Bozkurt B., Yigitoglu R. (2014). Influence of CPAP treatment on airway and systematic inflammation in OSAS patients. Sleep Breath..

[47] Bauça J.M., Yañez A., Fueyo L., de la Peña M., Pierola J., Sánchez-de-la-Torre A., Mediano O., Cabriada-Nuño V., Masdeu M.J., Duran-Cantolla J. (2017). Cell Death Biomarkers and Obstructive Sleep Apnea: Implications in the Acute Coronary Syndrome. Sleep.

[48] Morel F., Doussiere J., Vignais P.V. (1991). The superoxide-generating oxidase of phagocytic cells. Physiological, molecular and pathological aspects. JBIC J. Biol. Inorg. Chem..

[49] Zeng M.Y., Miralda I., Armstrong C.L., Uriarte S.M., Bagaitkar J. (2019). The roles of NADPH oxidase in modulating neutrophil effector responses. Mol. Oral Microbiol..

[50] Lemineur T., Deby-Dupont G., Preiser J.C. (2006). Biomarkers of oxidative stress in critically ill patients: What should be measured, when and how?. Curr. Opin. Clin. Nutr. Metab. Care.

[51] Liu H.G., Liu K., Zhou Y.N., Xu Y.J. (2009). Relationship between reduced nicotinamide adenine dinucleotide phosphate oxidase subunit p22phox gene polymorphism and obstructive sleep apnea-hypopnea syndrome in the Chinese Han population. Chin. Med. J..

[52] Soccio M., Toniato E., Evangelista V., Carluccio M., De Caterina R. (2005). Oxidative stress and cardiovascular risk: The role of vascular NAD(P)H oxidase and its genetic variants. Eur. J. Clin. Investig..

[53] Piérola J., Alemany A., Yañez A.M., de la Peña M., Sánchez-de-la-Torre M., Esquinas C., Pérez-Gutierrez C., Burguera B., Barbé F., Barceló A. (2011). NADPH oxidase p22phox polymorphisms and oxidative stress in patients with obstructive sleep apnoea. Respir. Med..

[54] Di Castelnuovo A., Soccio M., Iacoviello L., Evangelista V., Consoli A., Vanuzzo D., Diviacco S., Carluccio M., Rignanese L., De Caterina R. (2008). The C242T polymorphism of the p22phox component of NAD(P)H oxidase and vascular risk. Two case-control studies and a meta-analysis. Thromb. Haemost..

[55] Zhan G., Serrano F., Fenik P., Hsu R., Kong L., Pratico D., Klann E., Veasey S.C. (2005). NADPH oxidase mediates hypersomnolence and brain oxidative injury in a murine model of sleep apnea. Am. J. Respir. Crit. Care Med..

[56] Nair D., Dayyat E.A., Zhang S.X., Wang Y., Gozal D. (2011). Intermittent hypoxia-induced cognitive deficits are mediated by NADPH oxidase activity in a murine model of sleep apnea. PLOS ONE.

[57] Loffredo L., Zicari A.M., Occasi F., Perri L., Carnevale R., Angelico F., Del Ben M., Martino F., Nocella C., Savastano V. (2015). Endothelial dysfunction and oxidative stress in children with sleep disordered breathing: Role of NADPH oxidase. Atherosclerosis.

[58] Schulz R., Murzabekova G., Egemnazarov B., Kraut S., Eisele H.J., Dumitrascu R., Heitmann J., Seimetz M., Witzenrath M., Ghofrani H.A. (2014). Arterial hypertension in a murine model of sleep apnea: Role of NADPH oxidase 2. J. Hypertens..

[59] Lavie L., Vishnevsky A., Lavie P. (2004). Evidence for lipid peroxidation in obstructive sleep apnea. Sleep.

[60] Alzoghaibi M.A., Bahammam A.S. (2011). The effect of one night of continuous positive airway pressure therapy on oxidative stress and antioxidant defense in hypertensive patients with severe obstructive sleep apnea. Sleep Breath..

[61] Oyama J., Yamamoto H., Maeda T., Ito A., Node K., Makino N. (2012). Continuous positive airway pressure therapy improves vascular dysfunction and decreases oxidative stress in patients with the metabolic syndrome and obstructive sleep apnea syndrome. Clin. Cardiol..

[62] Caimi G., Montana M., Canino B., Calandrino V., Lo Presti R., Hopps E. (2016). Erythrocyte deformability, plasma lipid peroxidation and plasma protein oxidation in a group of OSAS subjects. Clin. Hemorheol. Microcirc..

[63] Mar H.L.P.Y., Hazen S.L., Tracy R.P., Strohl K.P., Auckley D., Bena J., Wang L., Walia H.K., Patel S.R., Mehra R. (2016). Effect of Continuous Positive Airway Pressure on Cardiovascular Biomarkers: The Sleep Apnea Stress Randomized Controlled Trial. Chest.

[64] Papandreou C. (2013). Levels of TBARS are inversely associated with lowest oxygen saturation in obese patients with OSAS. Sleep Breath..

[65] Stradling J., Schwarz E.I., Schlatzer C., Manuel A., Lee R., Antoniades C., Kohler M. (2015). Biomarkers of oxidative stress following continuous positive airway pressure withdrawal: Data from two randomised trials. Eur. Respir. J..

[66] Fernandez Alvarez R., Rubinos Cuadrado G., Alonso Arias R., Cascon Hernandez J.A., Palomo Antequera B., Iscar Urrutia M., Casan Clara P. (2016). Snoring as a Determinant Factor of Oxidative Stress in the Airway of Patients with Obstructive Sleep Apnea. Lung.

[67] Villa M.P., Supino M.C., Fedeli S., Rabasco J., Vitelli O., Del Pozzo M., Gentile G., Lionetto L., Barreto M., Simmaco M. (2014). Urinary concentration of 8-isoprostane as marker of severity of pediatric OSAS. Sleep Breath..

[68] Murri M., Alcázar-Ramírez J.D., Garrido-Sánchez L., Linde F., Alcaide J., Cardona F., Tinahones F.J. (2009). Oxidative stress and metabolic changes after continuous positive airway pressure treatment according to previous metabolic disorders in sleep apnea-hypopnea syndrome patients. Transl. Res..

[69] Witko-Sarsat V., Friedlander M., Capeillère-Blandin C., Nguyen-Khoa T., Nguyen A.T., Zingraff J., Jungers P., Descamps-Latscha B. (1996). Advanced oxidation protein products as a novel marker of oxidative stress in uremia. Kidney Int..

[70] He Y., Chen R., Wang J., Pan W., Sun Y., Han F., Wang Q., Liu C. (2016). Neurocognitive impairment is correlated with oxidative stress in patients with moderate-to-severe obstructive sleep apnea hypopnea syndrome. Respir. Med..

[71] Passali D., Corallo G., Petti A., Longini M., Passali F.M., Buonocore G., Bellussi L.M. (2016). A comparative study on oxidative stress role in nasal breathing impairment and obstructive sleep apnoea syndrome. Studio comparativo sul ruolo dello stress ossidativo nei pazienti con insufficienza respiratoria nasale e sindrome delle apnee ostruttive notturne. Acta Otorhinolaryngol. Ital..

[72] Zhou L., Chen P., Peng Y., Ouyang R. (2016). Role of Oxidative Stress in the Neurocognitive Dysfunction of Obstructive Sleep Apnea Syndrome. Oxidative Med. Cell. Longev..

[73] Pialoux V., Hanly P.J., Foster G.E., Brugniaux J.V., Beaudin A.E., Hartmann S.E., Pun M., Duggan C.T., Poulin M.J. (2008). Effect of 4 days of intermittent hypoxia on oxidative stress in healthy men. FASEB J..

[74] Özben S., Huseyinoglu N., Hanikoglu F., Güvenç T.S., Yildirim B.Z., Cort A., Özdem S., Ozben T., Yıldırım B.Z. (2014). Advanced oxidation protein products and ischaemia-modified albumin in obstructive sleep apnea. Eur. J. Clin. Investig..

[75] Tóthová L., Hodosy J., Mucska I., Celec P. (2014). Salivary markers of oxidative stress in patients with obstructive sleep apnea treated with continuous positive airway pressure. Sleep Breath..

[76] Tóthová Ľ., Celec P., Mucska I., Hodosy J. (2019). Short-term effects of continuous positive airway pressure on oxidative stress in severe sleep apnea. Sleep Breath..

[77] Khan N., Lambert-Messerlian G., Monteiro J.F., Hodosy J., Tóthová Ľ., Celec P., Eklund E., Curran P., Bourjeily G. (2017). Oxidative and carbonyl stress in pregnant women with obstructive sleep apnea. Sleep Breath..

[78] Galeazzi M., Morozzi G., Piccini M., Chen J., Bellisai F., Fineschi S., Marcolongo R. (2003). Dosage and characterisation of circulating DNA: Present usage and possible applications in systemic autoimmune disorders. Autoimmun. Rev..

[79] Bakan E., Fidan V., Alp H.H., Baygutalp N.K., Cokluk E. (2015). Effect of Modified Fujita Technique Uvulopalatoplasty on Oxidative DNA Damage Levels in Patients with Obstructive Sleep Apnea Syndrome. J. Craniofacial Surg..

[80] Ermakov A.V., Konkova M.S., Kostyuk S.V., Izevskaya V.L., Baranova A., Veiko N.N. (2013). Oxidised extracellular DNA as a stress signal in human cells. Oxidative Med. Cell. Longev..

[81] Lo Y.M., Tein M.S., Lau T.K., Haines C.J., Leung T.N., Poon P.M., Wainscoat J.S., Johnson P.J., Chang A.M., Hjelm N.M. (1998). Quantitative analysis of fetal DNA in maternal plasma and serum: Implications for noninvasive prenatal diagnosis. Am. J. Hum. Genet..

[82] Yamauchi M., Nakano H., Maekawa J., Okamoto Y., Ohnishi Y., Suzuki T., Kimura H. (2005). Oxidative stress in obstructive sleep apnea. Chest.

[83] Jurado-Gámez B., Fernandez-Marin M.C., Gómez-Chaparro J.L., Muñoz-Cabrera L., Lopez-Barea J., Perez-Jimenez F., Lopez-Miranda J. (2010). Relationship of oxidative stress and endothelial dysfunction in sleep apnoea. Eur. Respir. J..

[84] Testelmans D., Tamisier R., Barone-Rochette G., Baguet J.P., Roux-Lombard P., Pépin J.L., Lévy P. (2013). Profile of circulating cytokines: Impact of OSA, obesity and acute cardiovascular events. Cytokine.

[85] Li K., Wei P., Qin Y., Wei Y. (2017). Is C-reactive protein a marker of obstructive sleep apnea? A meta-analysis. Medicine.

[86] Chen C.Y., Chen C.L., Yu C.C., Chen T.T., Tseng S.T., Ho C.H. (2015). Association of inflammation and oxidative stress with obstructive sleep apnea in ischemic stroke patients. Sleep Med..

[87] Arnardottir E.S., Maislin G., Schwab R.J., Staley B., Benediktsdottir B., Olafsson I., Juliusson S., Romer M., Gislason T., Pack A.I. (2012). The interaction of obstructive sleep apnea and obesity on the inflammatory markers C-reactive protein and interleukin-6: The Icelandic Sleep Apnea Cohort. Sleep.

[88] Nadeem R., Molnar J., Madbouly E.M., Nida M., Aggarwal S., Sajid H., Naseem J., Loomba R. (2013). Serum inflammatory markers in obstructive sleep apnea: A meta-analysis. J. Clin. Sleep Med..

[89] Campos-Rodriguez F., Asensio-Cruz M.I., Cordero-Guevara J., Jurado-Gamez B., Carmona-Bernal C., Gonzalez-Martinez M., Troncoso M.F., Sanchez-Lopez V., Arellano-Orden E., Garcia-Sanchez M.I. (2019). Effect of continuous positive airway pressure on inflammatory, antioxidant, and depression biomarkers in women with obstructive sleep apnea: A randomised controlled trial. Sleep.

[90] Brånén L., Hovgaard L., Nitulescu M., Bengtsson E., Nilsson J., Jovinge S. (2004). Inhibition of tumor necrosis factor-alpha reduces atherosclerosis in apolipoprotein E knockout mice. Arter. Thromb. Vasc. Biol..

[91] Li Q., Zheng X. (2017). Tumor necrosis factor alpha is a promising circulating biomarker for the development of obstructive sleep apnea syndrome: A meta-analysis. Oncotarget.

[92] Vgontzas A.N., Zoumakis E., Lin H.M., Bixler E.O., Trakada G., Chrousos G.P. (2004). Marked decrease in sleepiness in patients with sleep apnea by etanercept, a tumor necrosis factor-alpha antagonist. J. Clin. Endocrinol. Metab..

[93] McNicholas W.T. (2009). Obstructive sleep apnea and inflammation. Prog. Cardiovasc. Dis..

[94] Sahlman J., Miettinen K., Peuhkurinen K., Seppä J., Peltonen M., Herder C., Punnonen K., Vanninen E., Gylling H., Partinen M. (2009). The activation of the inflammatory cytokines in overweight patients with mild obstructive sleep apnoea. J. Sleep Res..

[95] Williams R., Lemaire P., Lewis P., McDonald F.B., Lucking E., Hogan S., Sheehan D., Healy V., O’Halloran K.D. (2015). Chronic intermittent hypoxia increases rat sternohyoid muscle NADPH oxidase expression with attendant modest oxidative stress. Front. Physiol..

[96] In E., Özdemir C., Kaman D., Sökücü S.N. (2015). Heat Shock Proteins, L-Arginine, and Asymmetric Dimethylarginine Levels in Patients With Obstructive Sleep Apnea Syndrome. Arch. Bronconeumol..

[97] Zinellu A., Fois A.G., Mangoni A.A., Paliogiannis P., Sotgiu E., Zinellu E., Marras V., Pirina P., Carru C. (2018). Systemic concentrations of asymmetric dimethylarginine (ADMA) in chronic obstructive pulmonary disease (COPD): State of the art. Amino Acids.

[98] Eisele H.J., Markart P., Schulz R. (2015). Obstructive Sleep Apnea, Oxidative Stress, and Cardiovascular Disease: Evidence from Human Studies. Oxidative Med. Cell. Longev..

[99] Wysocka E., Cofta S., Cymerys M., Gozdzik J., Torlinski L., Batura-Gabryel H. (2008). The impact of the sleep apnea syndrome on oxidant-antioxidant balance in the blood of overweight and obese patients. J. Physiol. Pharmacol. Off. J. Pol. Physiol. Soc..

[100] Singh T.D., Patial K., Vijayan V.K., Ravi K. (2009). Oxidative stress and obstructive sleep apnoea syndrome. Indian J. Chest Dis. Allied. Sci..

[101] Barceló A., Barbé F., de la Peña M., Vila M., Pérez G., Piérola J., Durán J., Agustí A.G. (2006). Antioxidant status in patients with sleep apnoea and impact of continuous positive airway pressure treatment. Eur. Respir. J..

[102] Phillips C.L., Grunstein R.R. (2006). Obstructive sleep apnoea: Time for a radical change?. Eur. Respir. J..

[103] Shahar E., Whitney C.W., Redline S., Lee E.T., Newman A.B., Nieto F.J., O’Connor G.T., Boland L.L., Schwartz J.E., Samet J.M. (2001). Sleep-disordered breathing and cardiovascular disease: Cross-sectional results of the Sleep Heart Health Study. Am. J. Respir. Crit. Care Med..

[104] Johnson K.G., Johnson D.C. (2010). Frequency of sleep apnea in stroke and TIA patients: A meta-analysis. J. Clin. Sleep Med..

[105] McEvoy R.D., Antic N.A., Heeley E., Luo Y., Ou Q., Zhang X., Mediano O., Chen R., Drager L.F., Liu Z. (2016). CPAP for prevention of cardiovascular events in obstructive sleep apnea. N. Engl. J. Med..

[106] Peppard P.E., Young T., Barnet J.H., Palta M., Hagen E.W., Hla K.M. (2013). Increased prevalence of sleep-disordered breathing in adults. Am. J. Epidemiol..

[107] Narkiewicz K., van de Borne P.J., Pesek C.A., Dyken M.E., Montano N., Somers V.K. (1999). Selective potentiation of peripheral chemoreflex sensitivity in obstructive sleep apnea. Circulation.

[108] Narkiewicz K., Somers V.K. (2003). Sympathetic nerve activity in obstructive sleep apnoea. Acta Physiol. Scand..

[109] Imadojemu V.A., Mawji Z., Kunselman A., Gray K.S., Hogeman C.S., Leuenberger U.A. (2007). Sympathetic chemoreflex responses in obstructive sleep apnea and effects of continuous positive airway pressure therapy. Chest.

[110] Nanduri J., Peng Y.J., Wang N., Khan S.A., Semenza G.L., Kumar G.K., Prabhakar N.R. (2016). Epigenetic regulation of redox state mediates persistent cardiorespiratory abnormalities after long-term intermittent hypoxia. J. Physiol..

[111] Prabhakar N.R., Peng Y.J., Nanduri J. (2020). Hypoxia-inducible factors and obstructive sleep apnea. J. Clin. Investig..

[112] Treptow E., Pepin J.L., Bailly S., Levy P., Bosc C., Destors M., Woehrle H., Tamisier R. (2019). Reduction in sympathetic tone in patients with obstructive sleep apnoea: Is fixed CPAP more effective than APAP? A randomised, parallel trial protocol. BMJ Open.

[113] Wozniak D.R., Lasserson T.J., Smith I. (2014). Educational, supportive and behavioural interventions to improve usage of continuous positive airway pressure machines in adults with plo. Cochrane Database Syst. Rev..

[114] Maki-Nunes C., Toschi-Dias E., Cepeda F.X., Rondon M.U., Alves M.J., Fraga R.F., Braga A.M.F.W., Aguilar A.M., Amaro A.C., Drager L.F. (2015). Diet and exercise improve chemoreflex sensitivity in patients with metabolic syndrome and obstructive sleep apnea. Obesity.

[115] Liu L., Cao Q., Guo Z., Dai Q. (2015). Continuous Positive Airway Pressure in Patients With Obstructive Sleep Apnea and Resistant Hypertension: A Meta-Analysis of Randomized Controlled Trials. J. Clin. Hypertens..

[116] Hall A.B., Ziadi M.C., Leech J.A., Chen S.Y., Burwash I.G., Renaud J., deKemp R.A., Haddad H., Mielniczuk L.M., Yoshinaga K. (2014). Effects of short-term continuous positive airway pressure on myocardial sympathetic nerve function and energetics in patients with heart failure and obstructive sleep apnea: A randomised study. Circulation.

[117] Holmqvist F., Guan N., Zhu Z., Kowey P.R., Allen L.A., Fonarow G.C., Hylek E.M., Mahaffey K.W., Freeman J.V., Chang P. (2015). Impact of obstructive sleep apnea and continuous positive airway pressure therapy on outcomes in patients with atrial fibrillation-Results from the Outcomes Registry for Better Informed Treatment of Atrial Fibrillation (ORBIT-AF). Am. Hear. J..

[118] Kaneko Y., Floras J.S., Usui K., Plante J., Tkacova R., Kubo T., Ando S.-I., Bradley T.D. (2003). Cardiovascular effects of continuous positive airway pressure in patients with heart failure and obstructive sleep apnea. N. Engl. J. Med..

[119] Bradley T.D., Logan A.G., Kimoff R.J., Sériès F., Morrison D., Ferguson K., Belenkie I., Pfeifer M., Fleetham J., Hanly P. (2005). CANPAP Investigators. Continuous positive airway pressure for central sleep apnea and heart failure. N. Engl. J. Med..

[120] Kaushal N., Ramesh V., Gozal D. (2012). TNF-α and temporal changes in sleep architecture in mice exposed to sleep fragmentation. PLoS ONE.

[121] Wang Q., Wu Q., Feng J., Sun X. (2013). Obstructive sleep apnea and endothelial progenitor cells. Patient Prefer. Adherence.

[122] Li Y., Vgontzas A.N., Fernandez-Mendoza J., Kritikou I., Basta M., Pejovic S., Gaines J., Bixler E.O. (2016). Objective, but Not Subjective, Sleepiness is Associated With Inflammation in Sleep Apnea. Sleep.

[123] Kritikou I., Basta M., Vgontzas A.N., Pejovic S., Liao D., Tsaoussoglou M., Bixler E.O., Stefanakis Z., Chrousos G.P. (2013). Sleep apnoea, sleepiness, inflammation and insulin resistance in middle-aged males and females. Eur. Respir. J..

[124] Gottlieb D.J., Punjabi N.M., Mehra R., Patel S.R., Quan S.F., Babineau D.C., Tracy R.P., Rueschman M., Blumenthal R.S., Lewis E.F. (2014). CPAP versus oxygen in obstructive sleep apnea. N. Engl. J. Med..

[125] Stradling J.R., Craig S.E., Kohler M., Nicoll D., Ayers L., Nunn A.J., Bratton D.J. (2015). Markers of inflammation: Data from the MOSAIC randomised trial of CPAP for minimally symptomatic OSA [published correction appears in Thorax. 2015 Apr;70,319]. Thorax.

[126] Martin K., Stanchina M., Kouttab N., Harrington E.O., Rounds S. (2008). Circulating endothelial cells and endothelial progenitor cells in obstructive sleep apnea. Lung.

[127] Yun C.H., Jung K.H., Chu K., Kim S.H., Ji K.H., Park H.K., Kim H.C., Lee S.T., Lee S.K., Roh J.K. (2010). Increased Circulating Endothelial Microparticles and Carotid Atherosclerosis in Obstructive Sleep Apnea. J. Clin. Neurol..

[128] Iannella G., Magliulo G., Maniaci A., Meccariello G., Cocuzza S., Cammaroto G., Gobbi R., Sgarzani R., Firinu E., Corso R.M. (2020). Olfactory function in patients with obstructive sleep apnea: A meta-analysis study. Eur. Arch. Otorhinolaryngol..

[129] Chang C.P., Chia R.H., Wu T.L., Tsao K.C., Sun C.F., Wu J.T. (2003). Elevated cell-free serum DNA detected in patients with myocardial infarction. Clin. Chim. Acta.

